# Mis-Measuring Our Universities: Why Global University Rankings Don’t Add Up

**DOI:** 10.3389/frma.2021.680023

**Published:** 2021-09-09

**Authors:** Elizabeth Gadd

**Affiliations:** Research & Enterprise Office, Loughborough University, Loughborough, United Kingdom

**Keywords:** global rankings of universities, GDP, global inequities, responsible metrics, higher education institutions

## Abstract

Draws parallels between the problematic use of GDP to evaluate economic success with the use of global university rankings to evaluate university success. Inspired by Kate Raworth’s *Doughnut Economics,* this perspective argues that the pursuit of growth as measured by such indicators creates universities that ‘grow’ up the rankings rather than those which ‘thrive’ or ‘mature.’ Such growth creates academic wealth divides within and between countries, despite the direction of growth as inspired by the rankings not truly reflecting universities’ critical purpose or contribution. Highlights the incompatibility between universities’ alignment with socially responsible practices and continued engagement with socially irresponsible ranking practices. Proposes four possible ways of engendering change in the university rankings space. Concludes by calling on leaders of ‘world-leading’ universities to join together to ‘lead the world’ in challenging global university rankings, and to set their own standards for thriving and maturing universities.

## Introduction

In 2008, in the wake of the global financial crisis, President Nicolas Sarkozy commissioned senior economic thinkers Joseph Stiglitz, Amartya Sen and Jean Paul Fitoussi, to investigate how economic and social progress might better be measured. The resulting report, later published as a book called *Mis-Measuring Our Lives: Why GDP Doesn’t Add Up* (Stiglitz et al., 2010), concluded that the use of a single indicator to evaluate social progress was causing significant financial, economic, social, and environmental damage. These ideas have been developed more recently by Raworth (2017) in *Doughnut Economics*. In this perspective I argue that the same criticisms can be levelled at the use of global university rankings to assess the performance of higher education (HE) institutions and suggest some ways in which the HE community might seek to engender change.

## The Problem With Growth

The idea of growth is almost universally seen as a positive. However, as Raworth, Stiglitz, Sen and Fitoussi make clear, whether growth is actually a positive, depends on how you characterise and measure it. The use of a single indicator, Gross Domestic Product (GDP), to measure economic growth is hugely problematic because it largely ignores the means by which growth is achieved (including disregarding environmental consequences) and the impacts of that growth on the human race (such as growing inequality). Escalating numbers are living in poverty while wealth increasingly amasses in the hands of a few. This is the case even though GDP actually fails to capture all the elements that contribute to a growing economy (such as volunteering and childcare) and also fails to recognise that a growing economy does not represent everything that is important in life (such as fresh air and friendship). Despite this, society is politically locked into a GDP-based growth-at-all-costs mindset, because not to grow would be viewed as a failure, even though such growth might be leading to our own extinction.

Of course, higher education also increasingly operates as a market, much to the concern of many in the sector (Molesworth et al., 2010). If we accept that this is the case, then global university rankings are almost certainly its problematic single indicator of success. I say ‘single’ but in fact there are three dominant global rankings (Academic Ranking of World Universities (ARWU), Quacarelli-Simonds (QS) and Times Higher Education World University Rankings (THE WUR)), which share many similar features (see Bekhradnia (2016) for an overview) and many others besides (IREG, 2021).

University rankings share many characteristics with GDP. They are both single indicators seeking to signify the success of multi-faceted and hugely complex entities. They are both composite indicators which seek to incorporate different facets of those entities, but simultaneously fail to incorporate some of their vital qualities. Neither seek to normalise for inherited characteristics that give the entities measured an advantage over others (age, wealth and geography) and yet both provide their ‘winners’ with further advantages (membership of the G8 for example). Despite such criticisms, both established and emerging entities continue to trust GDP and university rankings as a benchmark even though, as the Sarcozy report put it, those attempting to do so “are like pilots trying to steer a course without a reliable compass.”

## Grow or Thrive?

One of the main justifications one hears for university rankings is that they enable HEIs in low and middle-income countries (LMICs) to leverage investment in HE where their governments otherwise might not do so. Indeed, in the last 20 years, Taiwan (Lu, 2004), Russia (Osipian, 2020), China (Anon, 2017) and Japan (Yonezawa, 2006) have all invested in programmes to develop ‘world-class’ (read ‘ranking-topping’) universities. Unfortunately, all too often that investment is made to enable institutions to climb the rankings rather than to develop strong universities (Munch, 2014). As Raworth observes about GDP, such indicators put pressure on entities to grow, whether or not they thrive. Whereas what we really need are indicators that cause entities to thrive, whether or not they grow. As newer entrants soon realise, unless they have the natural advantages of already highly ranked institutions (old, large, wealthy, ‘white,’ ‘Western,’ English-speaking research-intensives (Marginson and van der Wende, 2007; Salmi, 2009; University Wankings, 2021) their chances of displacing such organisations is very low. Thus, if they are unable to create a comparable university, their only option is to create a similar-looking surrogate.

To this end, we see university marketing budgets soaring as institutions seek to paint themselves as ‘world-leading’ (Moore et al., 2017; Hall and Weale, 2019). In India, a new class of ‘Institutions of Eminence’ has been created (India Ministry of Education, 2018), and recently this honour was bestowed on a new university, yet to prove its worth, perhaps in the hope that nominative determinism would do its work.

At the darker end of the spectrum of ranking-climbing activity, there are large numbers of HEIs seeking to either ‘game’ the rankings (Calderon, 2020) or simply to cheat. Activities might include legitimate attempts to solicit survey respondents that are likely to assess an HEI favourably or illegitimate practices such as paying for an institution’s name to appear on highly cited papers (Kehm, 2020), or “industrialised self-citation” activity to boost THE WUR citation scores (Holmes, 2017). Such activity is by no means limited to HEIs from LMICs, however. Morphew and Swanson (2011) report on activities by US universities to present admissions and faculty data in ways that are advantageous to their ranking position.

In some cases the rankings agencies themselves are seen to be complicit in gaming. Chirakov (2021) reports how Russian HEIs that frequently engage with QS Consultancy services seem to “improve their positions in global university rankings over time, regardless of improvements in their institutional quality,” observing that in the QS ranking, one's “faculty-student ratio score…can be “improved” relatively easily by adjusting the way data is reported.” Holmes (2016a) and Holmes (2016b) describe how changes to the calibration of THE WUR methodologies seem to favour the hosts of that year’s Times Higher Education Summits.

A last resort for institutions or regions that do not fare well in the existing rankings is to create their own. This was the origin of the ARWU ranking, developed by Shanghai Jao Tong University in an effort to challenge the dominance of Western universities. Recent efforts include Moscow’s Three University Missions Ranking (MosIUR, 2020) which puts six Russian universities in the top 200, outperforming the one that appears in the top 200 of the QS and THE WUR thus making headway on their otherwise failed ambition to get five institutions in the top 100 by 2020 (Osipian, 2020).

Of course, all this activity focuses energy and resource on developing universities that ‘grow’ up the rankings, rather than institutions that truly ‘thrive.’

## Grow or Mature?

It is not only emerging institutions that suffer at the hands of a growth (or climbing) fixation, it’s mature institutions too. This is because, as Raworth observes, nothing grows forever. In the natural world there is a growth phase followed by a maturing, fruit-bearing phase. Thus, when an institution matures, it would not be unusual for its income, productivity and other indicators currently assessed by global university rankings such as staff:student ratios to stabilise and with them, the institution’s rank.^1^ Indeed, the same could be observed of the wider academy. With the global rush to invest in research and development (R&D), questions are now being asked as to what is the optimum size of both individual HEIs and a nation’s R&D sector, and at what point does the return on investment start to diminish? However, whilst in the natural world, a plateauing of growth would be considered a healthy situation—a sign of a thriving entity—in the current HE economy, where success is measured by rank, this could have a significant negative impact on an institution’s long-term viability.

Instead, mature institutions, like mature economies, made anxious by this stasis, start taking drastic and desperate action in order to keep on climbing. Such actions might include merging smaller institutions into larger ones to increase their visibility and impact, as with the creation of France’s mega-university, Paris-Saclay (Anon, 2020). They might also involve dismissing researchers that fail to publish in highly-cited journals (Bonyhady and Chrysanthos, 2020) or recruiting only academics on Clarivate’s Highly Cited Researcher list (Baker, 2018). Just as unranked universities might develop a new ranking that better showcases their strengths, ranked universities might put a new spin on existing rankings that suggest they are higher than they are. A recent effort to aggregate the already aggregated scores from the three most prominent global rankings into the Aggregate Ranking of Top Universities (ARTU) by Australia’s University of New South Wales (2020) is a prime example.

Locke (2011) has observed that the global university rankings run on a deficit model, characterised by anxiety. Institutions are either anxious to be placed, anxious to climb, or anxious to retain their rank. However, it is those mature institutions at the top that have the most to lose; better to be on the rise than on the decline. Luckily for the ranking agencies, fear sells. As such there is no shortage of data and consultancy products available to those who wish to improve their position and the conflict of interests this represents has not gone unobserved (Chikorov, 2021). One such product is an exclusive club called the World 100 Reputation Network (2021) for institutions ranked in the top 200 of one of the big four global rankings to enable them to share strategies for retaining their ranking topping status.

## The Growing Inequity of Growing Inequity

This club is an excellent example of the Matthew effect (where the rich get richer, and the poor get poorer). Top 200 institutions have special status: funders fund them, talented students and faculty want to work at them and so governments invest in them. However, we know that it is the already wealthy, established, often English-speaking institutions in the global north that dominate the top 200. Their rank elevates their status, attracting greater endowments, enabling further investments in people and facilities, which further increases their lead. The effect of pursuing ranking-related ‘growth,’ just as with GDP, increasingly concentrates the ‘wealth’ (reputation and financial) in the hands of a few, leaving others without (Aldred, 2019).

Data from the OECD (2021) plotting global investment in tertiary eduction shows that in 1995 the spend per tertiary education student ranged from 0 to 15K USD, however by 2016 the range had almost doubled to 3.76K–30K USD. Whilst there may be many factors influencing these figures, two things are clear: 1) those at the top have stayed at the top, and 2) the disparity between the “haves” and the “have-nots” is growing, rather than shrinking.

Disparities within countries is as problematic as disparities between countries. As Sarkozy’s report points out, the use of averages to depict growth can mask huge inequities in the underlying data. Average income can go up, whilst the actual income of the majority of citizens goes down, obscured by the extremely high incomes of a small number of wealthy individuals inflating the mean.

There have not been many analyses of the growth of reputational or financial wealth of universities over time. However, an investigation by Shen (2013) demonstrated a growing disparity in academic salaries offered by the richer and poorer US universities. He showed that “a full professor at a public US doctoral university in 1970–71 could have expected a salary equal to 91% of what a colleague at a comparable private university earns. But by 2012–13, the proportion for a public university professor’s pay had declined to only 65% of his/her peers at private schools.”

A study exploring the geographical concentration of UK research funding recently showed that 49% of public R&D spend and 71% of capital infrastructure research spend between 2007 and 2014 was in London and the South-East of England—where the United Kingdom’s five top-ranked universities are based (Forth and Jones, 2020). A recent assessment of the impact of the COVID-19 pandemic on UK university finances showed that the thirteen in danger of insolvency were mainly less well-ranked universities more likely to be affected by a downturn in student recruitment (Drayton and Waltman, 2020).

Investments made by LMICs to get on the rankings’ ‘ladder’ are similarly concentrated around the small number of institutions where they feel they have the best chance of success. The consequence, as critics of India’s Institutions of Eminence point out, is that the rest of that nation’s higher education establishments get left behind (Mittal and Tiwari, 2020). Analyses of government-funded university excellence initiatives in other parts of the world such as China (Zong and Zang, 2019), Russia (Lovakov et al., 2021), and Japan (Yonezawa and Shimmi, 2015) all show considerably larger disparities between funded and unfunded institutions at the end of the exercise. These disparities are evident across a range of indicators such as publications in highly cited journals, international collaborations, and the recruitment of talented students and overseas academics.

It is for this reason that Hazelkorn (2017) suggests that governments should invest in world-class HE systems rather than world-class universities. While this still leads to global competitiveness, at least it promotes the funding of a broad range of HEIs that serve a range of local needs, rather than feeding some and starving others.

However, the problem is not just that some get left behind, but ultimately that the rankings they are climbing are not going to get them where they need to go. The pursuit of ranking-related ‘growth’ is at odds with the ability of universities to mature and thrive. This is because when you look at the behaviours necessary to climb the rankings, they are not behaviours that lead to healthier institutions, but ones that lead to toxic, unhappy institutions with deeply misplaced loyalties. Indeed, the dimensions evaluated by the global university rankings are not always representative of those that lead to a strong university at all.

## What Do Universities Actually Do?

The global rankings seek to assess universities across a number of dimensions: teaching, research, reputation, industry-focus, and collaboration. However, Selten et al. (2019) have demonstrated through principal component analysis and exploratory factor analysis that success in the rankings is essentially a function of an institution’s citations and reputation. Unfortunately, citations are a notoriously poor proxy for research quality (Gingras, 2014) and are measured by the rankings using bibliometric sources that significantly disadvantage the global south (Becerril-García, 2020).

Similarly, the use of reputation as a success indicator is hugely problematic. Firstly, reputation is never a true reflection of reality. As Abraham Lincoln once said, “Character is like a tree and reputation like its shadow. The shadow is what we think of it; the tree is the real thing.” Secondly, measuring a university’s reputation, like measuring shadows, is extremely difficult to do. Again, Selten et al. (2019) found that the opinion surveys used by the rankers to score a university’s reputation ultimately measured only brand awareness. Indeed, the THE WUR recently stated that they saw a university’s reputation as synonymous with its brand (Ross, 2021).

We can therefore conclude that the qualities ultimately measured by the global university rankings do not map onto the mission statements of most universities. Teaching and learning is a principal aim of all HEIs, and yet has no bearing on an institution’s rank. It is, of course, notoriously difficult to measure on a global scale and so rankers rely on very poor proxies such as staff:student ratios, alumni with Nobel prizes or a teaching reputation survey. Unfortunately, Selten et al. (2019) have demonstrated that teaching reputation surveys correlate closely with research reputation surveys, again suggesting that it is brand rather than teaching quality that is being measured.

Universities’ so-called ‘third missions’—their research impact and enterprise activity—are not measured at all by the mainstream university rankings. Lee et al. (2020) argue that this further discriminates against institutions in the global south that may be more mission-orientated. The THE WUR have recently introduced an Impact Ranking based on the UN Sustainable Development Goals, however, again due to the lack of globally comparable impact data, universities are largely left to supply their own evidence which does not make for an equitable comparison (Curry, 2020). Interestingly, this evidence is supplemented by more bibliographic data from the same globally skewed source as their mainstream ranking which rather mitigates against, rather than for, sustainable development.

However, even if the rankings were able to measure the quality of a university’s teaching, research and enterprise, evidence has shown that such successful outputs are largely a product of a university’s inputs: their age, wealth and geography. The university that has the wealth and reputation to recruit and resource the most talented academics is likely to get the best outcomes—especially when the world is pre-disposed to overvalue the outcomes of that already well-resourced and well-known university.

Such legacy “variables” should arguably be factored out of any truly responsible evaluation (Gadd et al., 2021). Indeed, what universities need to do to thrive and mature, and where all universities have an equal opportunity to succeed, is to create processes, policies and a culture that successfully convert their ‘inputs’ into their ‘outputs.’ The problem is that such things—the things we arguably value most about our universities: academic freedom, equality and diversity, good governance, and a positive teaching and research environment—are all largely unmeasurable.

## What to Do?

Whilst such critiques of global university rankings will not be new to any followers of the debate, what we have yet to see in response to two decades-worth of argument, is any real change in this space. The ranking agencies remain entirely unscathed by repeated criticism and continue to proliferate, whilst end-users seem impervious to their logic and continue to rely on the rankings as a lazy proxy for a university’s quality. As such institutions have had to accept global rankings as an established part of the HE landscape (the ‘rankings are here to stay’ narrative) and to promote their own rank in order to attract students, thus inadvertently lending the rankings legitimacy. In this way, rankings have become an uncontested institutional norm. Given that most HE institutions hold themselves to high standards around data transparency and openness which are not shared by the rankings, this is a particular irony.

It was against this backdrop that the INORMS Research Evaluation Working Group sought to consolidate best practice in the field of ‘responsible’ university rankings in the form of a set of principles, and to highlight the extent to which the most high-profile rankings met those criteria. They all fell short, and the most high-profile rankings fell even more short than the others. This work has been widely publicised (INORMS, 2020; Gadd et al., 2021), including a piece in Nature (Gadd, 2020), however, to date there has been no response—formal or informal—from the ‘big three’ global rankings (ARWU, QS and THE WUR). It should be noted that other rankings such as the Leiden Ranking and U-Multirank fared much better against the INORMS principles. However, ironically, whilst not seeking to identify the world’s ‘top’ institutions overall won them higher scores on the INORMS ratings, this diminishes their influence globally as end-users prize quick and easy answers, even if inaccurate.

The question then remains as to how to initiate change in this domain when the key stakeholders are, like those organisations at the top of their rankings, wealthy, powerful, and seemingly impervious to critique. Are there lessons we can learn from the more long-standing and parallel problem posed by the use of GDP to measure economic success?

## Independent Regulation

One of the challenges of university rankings is that they are self-appointed and unaccountable. The International Rankings Expert Group (IREG, 2021) claims to be an “independent” body offering ranking audits, however, a large proportion of the seats on their executive committee are occupied by ranking agencies themselves. Were the rankings overseen by a truly independent body, just as the calculation of GDP is overseen by national statistical offices around the world which report into the UN Statistical Authority, this might provide a useful challenge to some of their methodological deficiencies. An obvious choice would be the Royal Statistical Society (RSS), an international organisation whose mission includes campaigning for the effective use of statistics for the public good. The RSS recently turned their attention to the United Kingdom Teaching Excellence Framework on the grounds that it was “likely to mislead students who use TEF to inform their university choices” (Royal Statistical Society, 2019). The global university rankings as currently formulated are clearly subject to the same accusation, and a rigorous investigation by such a prestigious and independent body could be enormously influential.

## Start a New Game?

Another option for challenging the dominance of an existing unhelpful indicator, as Raworth suggests, is to introduce an alternative. She describes the Human Development Index (UNDP, 2021), a dashboard of alternative indicators to GDP, which measures dimensions such as long life, education and living standards, which can lead to positive societal change. Of course, there is no shortage of challengers to the dominance of the current input/output dominated world rankings. Some are serious, such as the Universitas Indonesia (2020) Green University Rankings, others are less so (Greatrix, 2020).

The problem with new indicators is that all too often they do not displace existing ones but at best complement them and at worst are completely overshadowed by them. However, if the heavy users of such rankings, such as research studentship funders, could collectively agree to focus on indicators that the HE community agree are a better representation of their contribution, then this could be a significant step forward. For just as ranking agencies seek to exploit the marketplace that is Higher Education, they too are subject to the demands of that marketplace. Should the demand for their services change, their influence would change with it. It is this thought that leads to my third suggestion.

## Leaders Lead

Whilst critiques of global university rankings are not new, what I believe is new, as the appetite for Raworth’s *Doughnut Economics* has shown, is our unwillingness to tolerate initiatives that no longer align with our principles and that lead to poor outcomes for our planet and our people. The world has changed from one in which we turn a blind eye to inconvenient truths to one where we seek to tackle them head on.

In the last 10 years we have seen a growth in public statements of commitment to socially responsible practices by corporates, charities and publicly funded organisations alike. In Higher Education there has been a spotlight on Equity, Diversity and Inclusion (EDI), sustainability, improving research culture, Responsible Research & Innovation (RRI), open research and of course responsible research evaluation. Universities have declared their commitment to responsible practices through accreditation with organisations like Athena Swan (Advance HE, 2021), Stonewall (2021), the Race Equality Charter (Advance HE, 2020), the UK Reproducibility Network (UKRN, 2021), and through adopting principles such as those espoused by the Declaration on Research Assessment (DORA, 2021), the Leiden Manifesto (Hicks et al., 2015) or the Hong Kong Principles on Researcher Evaluation (Moher et al., 2020).

When one considers the perverse effects of the global university rankings: their deeply problematic methodologies that lead to a pursuit of “growing” rather than “thriving” or “maturing”; their bias towards already established, wealthy, English-speaking organisations in the global north; and their contribution towards growing academic inequities across and within countries; it is hard to understand how an organisation that is truly committed to responsible research evaluation and other socially responsible practices can legitimately continue to engage with them.

Of course, one has sympathy with divided leaders who are fully cognizant of the rankings’ flaws whilst simultaneously having to rely on them to survive in a HE marketplace that is not of their making. However, in a world where leaders are increasingly called upon to make hard value-led choices, we may be approaching a time where these fundamentally incompatible positions cannot be maintained. As Leeds University’s Vice Chancellor, Simone Buitendijk, recently observed.

*“If there was ever a good time to define the moral narrative for global institutions’ strategies, whether businesses, NGOs or universities, it is now. COVID has taught us the importance of prioritising human values over competition for profits, or for limited, metricised and quantitative outcomes”* (Buitendijk, 2021).

There is currently an opportunity for HEIs to rethink both participation in, and promulgating the results of, global university rankings that better aligns with institutional values. Indeed, the (European Commission’s, 2020) recent report *Towards a 2030 Vision on the Future of Universities in Europe* directly challenged the reliance on university rankings as an “overly simplistic” measure of university success, preferring alternative metrics that highlight universities’ wider contribution. I would suggest that this report may provide a key as to how leaders might operationalise any move to challenge the unwelcome impacts of the global university rankings, namely, as a collective.

## Collective Action

Growth addiction can only be challenged by those who have grown: those institutions well-served by the current system. As Masood (2016) observed about GDP, “Any revision to the index won’t pass muster unless the interests of its founder countries are protected…permanent members of the UN Security Council will not allow a change to GDP that leads to them slipping down the league table.”

Given that the global university rankings make rivals of those entities, the only real way they are going to successfully change the system is if they join forces and agree to challenge it together. We see an example of this in the C40 (2021) network of 80 megacities (representing 25% of world GDP) who are collaborating to tackle climate change.

If senior leaders of so-called ‘world-leading’ mission- and value-led institutions are serious about delivering on their mission and values, it would seem logical that instead of joining exclusive World 100 reputation networks that keep less advantaged institutions from poorer countries out, they should create open, outward-facing networks that let such institutions in. As Gloria Steinem famously said, “Imagine we are linked, not ranked.” Were universities depicted in terms of a network, rather than a ranking, it might reinforce the fact that this is a group of organisations with the same mission, and not a group of organisations in the same competition. Whilst institutions may not have the power to prevent third parties from ranking them, they do have the power to self-characterise themselves, and to act, as a network, and not a network that collaborates only in order to compete, one but that collaborates to do good in the world.

Instead of perpetuating the myth that global university rankings measure those things that create strong, thriving institutions, we need a new breed of principled, connected university leaders to actively call them out for their poor, Matthew effect-inducing methodologies, who commit not to use them as KPIs, provide them with data, mention them in marketing, and to avoid ranking-organised summits that further legitimise them. I am also aware this will require persuading their own governing bodies not to offer bonuses based on their rank (Musselin, 2018). Perhaps in an extension of the ‘I am not my h-index’ campaign promoted by researchers (Curry, 2018), we need a new campaign for universities called, “So much more than our rank”?

To be clear, this is not about giving up on notions of excellence or quality, it is about university leaders being the ones who get to define what those notions mean. It is also about saying no to the scarcity mindset generated by the global rankings, in a world where there is enough to go round.

I accept that this kind of action is on a different scale to anything previously seen in the responsible research assessment space. It has been relatively painless for institutions to implement DORA or the Leiden Manifesto—some adjustments to internal policy and process were all that was needed. The collective will required to challenge the negative impacts of the reputation-based economy as measured by the current world university rankings, necessitates looking beyond our own institutions and scrutinising their long-term, systemic, global effects. As Roman Krznaric (2021) reminds us in *The Good Ancestor: Long-Term Thinking in a Short-Term World*, we need to make the decisions now that our descendants will thank us for. Such perspectives are not often prioritised by HE administrators. However, the tide might be turning.

Dame Ottoline Leyser, CEO of UK Research & Innovation (UKRI) has started to promote the notion of ‘net contribution’ in the research arena: a suggestion that we are rewarded not only for the contribution we make, but for the contribution we enable others to make (Leyser, 2020). If this approach is more widely adopted it might encourage a broader definition of university ‘success’—because another’s success, and your contribution to it, becomes your success.

I am presenting here the moral argument, of course, because these are the claims that universities are starting to make for themselves. However, there is a pragmatic argument too. For just as the logical extension of a GDP-based growth addiction is a society where there is not enough disposable income amongst the general population to purchase the products and services of the wealthy few, so will pitting universities against one another in a global competition where only a few similar-looking institutions survive, eventually impoverish us all. We need a diversity of flourishing higher education institutions that serve the diverse needs and developmental stages of the world we inhabit if we are to thrive as a human race. If the current global crises have taught us anything it is, as the thought-leading Margaret Heffernan (2014) points out, that “no one wins unless everybody wins.”

If institutions are genuinely committed to responsible evaluation practice, to equity, diversity, and inclusion, and if they are genuinely committed to delivering on their own mission to positively impact the world with their teaching and research, I would argue that this is incompatible with overlooking the negative impacts of the global university rankings.

As Raworth observed about GDP, it is time to move from “economic thinking” to “economic doing.” I would urge the senior leaders of any institution that considers itself to be world-leading to lead the world in this significant and important matter. They can do so by joining forces with other principled leaders to proactively stand against substandard notions of excellence and harmful forms of competition that neither reflect their own contribution nor the contribution of their mission-sharing global network. Instead, I encourage them to work with that network to redefine what a thriving and maturing university does, namely, to develop mission-specific policies, processes and cultures that achieve their important ends, and endorse efforts to evaluate them accordingly.

## Data Availability

The original contributions presented in the study are included in the article further inquiries can be directed to the corresponding author.

## Footnotes

1 Of course, one of the failings of rankings is that they count things like Nobel prizes from time immemorial, giving some older institutions an eternal advantage.
